# Supplementary material for: Content-rich biological network constructed by mining PubMed abstracts
Source: BMC Bioinformatics. 2004 Oct 8;5:147. doi: 10.1186/1471-2105-5-147 (PMC528731; doi:10.1186/1471-2105-5-147)
Supplement: Additional File 2 — The original results of the above study (non-essential files are deleted to keep the file size under the limit set by BMC bioinformatics). [file 1471-2105-5-147-S2.bz2 › chilibotAdditionalFile2/dip10/749ID10485884E18132/html/BTUB_TONB.html]

 


 **BTUB** and **TONB** 
  
Found 21 abstracts in PubMed, retrieved 10.  
 

 What does Google say? 
 PDF only 
| .edu only 

---

**Interactive relationship** (e.g. stimulation, inhibition, etc)

**Inhibitory relationship**- This inhibition by CN Cbl is increased when the expression of FhuA and  **TonB**  is repressed by growth with excess iron and is eliminated when  **BtuB**  synthesis is repressed by CN Cbl.  Ref: 7665457 J Bacteriol, 1995
- Here, site directed disulfide crosslinking demonstrates contact between a conserved region of  **BtuB** , called the  **TonB**  box, and a portion of  **TonB** , previously implicated as the site of suppressors of  **TonB**  box mutations.  Ref: 10485884 Proc Natl Acad Sci U S A, 1999
- The Ton box sequence near the amino terminus of  **BtuB**  is conserved among all  **TonB**  dependent transporters and is the only known site of mutations that confer a transport defective phenotype which can be suppressed by certain substitutions at residue 160 in  **TonB** .  Ref: 11029413 J Bacteriol, 2000
**Neutral relationship**- Site directed disulfide bonding reveals an interaction site between energy coupling protein  **TonB**  and  **BtuB** , the outer membrane cobalamin transporter.  Ref: 10485884 Proc Natl Acad Sci U S A, 1999
- Additional evidence for interaction of the Cbl and iron transport systems is provided by the strong stimulation of the  **BtuB**  and  **TonB**  dependent transport of CN Cbl into a nonexchangeable, presumably cytoplasmic pool by preincubation of cells with the iron II chelator dipyridyl.  Ref: 7665457 J Bacteriol, 1995
- Sequence changes in the ton box region of  **BtuB**  affect its transport activities and interaction with  **TonB**  protein.  Ref: 11029413 J Bacteriol, 2000
- This interaction with  **TonB**  occurs in part through an N terminal segment in the  **BtuB**  sequence called the Ton box.  Ref: 11705387 Biochemistry, 2001
- Uptake of cobalamins by the transporter protein  **BtuB**  in the outer membrane of Escherichia coli requires the proton motive force and the transperiplasmic protein  **TonB** .  Ref: 11029413 J Bacteriol, 2000
- The bacterial outer membrane transporter for vitamin B 12,  **BtuB** , derives its energy for transport by interacting with the trans periplasmic membrane protein  **TonB** .  Ref: 11705387 Biochemistry, 2001
- These data are summarized on a two dimensional folding model for the  **BtuB**  protein in the outer membrane devised using both amphipathic beta strand analysis and sequence conservation amongst the  **TonB**  dependent receptors.  Ref: 7746157 Mol Microbiol, 1995
- The uncoupled proline substitutions in  **BtuB**  markedly affected the pattern of disulfide bonding to  **TonB** , both increasing the extent of cross linking and shifting the pairs of residues that can be joined.  Ref: 11029413 J Bacteriol, 2000
- Site directed disulfide bonding in intact cells has been used to show that the Ton box of  **BtuB**  and residues around position 160 of  **TonB**  approach each other in a highly oriented and specific manner to form  **BtuB**   **TonB**  heterodimers that are stimulated by the presence of transport substrate.  Ref: 11029413 J Bacteriol, 2000

**Non-interactive relationship** (e.g. studied together, co-existance, homology, etc.)

- Transport of vitamin B 12 across the outer membrane of Escherichia coli, like that of iron siderophore complexes, is an active transport process requiring a specific outer membrane transporter  **BtuB** , the proton motive force, and the trans periplasmic energy coupling protein  **TonB** .  Ref: 10485884 Proc Natl Acad Sci U S A, 1999
- Cross linking of  **BtuB**  and  **TonB**  in the presence of the  **BtuB**  V10P substitution became independent of the presence of substrate, indicating an additional distortion of the exposure of the Ton box in the periplasmic space.  Ref: 11029413 J Bacteriol, 2000
- The defect in cobalamin utilization resulting from the L8P substitution was suppressed by cysteine substitutions in adjacent residues in  **BtuB**  or in  **TonB** .  Ref: 11029413 J Bacteriol, 2000
- Transport of CN Cbl across the outer membrane and its accumulation in the periplasm is mediated by the  **TonB**  dependent transporter  **BtuB** .  Ref: 11790740 J Bacteriol, 2002
- We consider a variety of different colicins, including A, B, D, E1, E3, Ia, M and N, that penetrate through the porins OmpF, FepA,  **BtuB** , Cir and FhuA, to subsequently interact with a few targets in the periplasm, including TolA, TolB, TolC and  **TonB** .  Ref: 12423783 Biochimie< MedlineTA>Biochimie, 2002
- Crosslinking at several positions was increased when  **BtuB**  was loaded with substrate, and the crosslinking pattern was altered by the presence of substitutions in  **BtuB**  that cause a  **TonB**  uncoupled phenotype.  Ref: 10485884 Proc Natl Acad Sci U S A, 1999
- In a transport defective mutant of  **BtuB** , this conformational cycle is disrupted and the  **TonB**  box appears to be extended even in the absence of substrate.  Ref: 10700278 Nat Struct Biol, 2000
